# Pharmacokinetics of biologics in gastric cancer

**DOI:** 10.1111/cts.13474

**Published:** 2023-01-18

**Authors:** Junyi Li, David C. Turner, Feifei Li, Xi Chen, Michael Z. Liao, Chunze Li

**Affiliations:** ^1^ Genentech Inc. South San Francisco California USA; ^2^ GSK Upper Providence Pennsylvania USA

## Abstract

Gastric cancer (GC) remains one of the leading causes of cancer death worldwide despite improvements in therapeutic options. Several biologics have been investigated in patients with GC, including those approved in other solid tumors; however, the success rate of the pivotal trials that investigated these biologic molecules in GC remains low. Elevation in total clearance and a decrease in systemic pharmacokinetic (PK) exposure in GC compared with other indications have been observed in these biologics across different pathways. Achieving optimal exposure for patients with GC is an important factor in balancing risk and optimizing therapeutic benefit and thus maximizing chance of positive outcomes for pivotal trials. Therefore, in this review, we summarize the PK disposition of several molecules (e.g., anti‐HER2, anti‐VEGF, and anti‐PD1) evaluated in GC and showed a consistent trend of lower drug exposure as compared to other solid tumors. We hypothesize that two possible mechanisms: (1) hyper‐catabolism of endogenous and exogenous proteins due to cancer cachexia; and (2) gastric protein leakage due to local inflammation at the gastrointestinal tract may explain or partially explain the increase of clearance in patients with GC. Last, the potential implications of such findings on dose selection to optimize the benefit: risk profile for biologics in GC are also discussed.

## INTRODUCTION

Gastric cancer (GC), including adenocarcinoma of the gastroesophageal junction (GEJ) and stomach remains one of the leading causes of cancer death worldwide, especially in East Asia. Although both incidence and death rates within the United States have decreased in the last decades, GC still accounts for about 1.5% of all new cancers diagnosed in the United States each year, and the overall 5‐year relative survival rate remains at 32.4%.^1^ Surgery is still regarded as the only curative therapy, whereas peri‐operative and adjuvant chemotherapy, as well as chemoradiation, remain the front‐line therapy to improve overall survival and quality of life.

Advancements in target identification and antibody engineering have led to several recent approvals of targeted therapies for advanced GC. Currently, five biologics are approved for treatment of GC: Herceptin (trastuzumab) and Enhertu (trastuzumab deruxtecan) which target human epidermal growth factor receptor 2 (HER2), Keytruda (pembrolizumab) and Opdivo (nivolumab) which target programmed death 1 (PD‐1), and Cyramza (ramucirumab) which targets vascular endothelial growth factor receptor (VEGFR). However, among more than 40 phase II/III clinical trials for over 28 molecules in advanced GC completed by 2019, only about one third showed positive results.^2^ Several molecules, including those which share the same targets as approved in other indications (e.g., pertuzumab and trastuzumab emtansine target HER2, bevacizumab targets VEGFR) have shown negative results in their respective pivotal clinical trials for GC.

Elevated clearance (CL) and decreased pharmacokinetic (PK) exposure in GC have been observed across biologics that target different mechanisms of action (Table 1). These consistent observations have prompted some concern as to whether an optimal dose was tested in the pivotal trials, especially if the dose tested in GC was based on other indications. It is important to leverage disease biology, dose/exposure, and its relationship with response to ensure an optimal dose is selected to ensure positive clinical outcomes. In this review, we summarize the PK disposition for several monoclonal antibodies (mAbs)/antibody‐drug conjugates (ADCs) that have been evaluated in GC and other solid tumors. We also discuss several mechanisms by which the PKs of these biologics may be altered in patients with GC, which include both target‐related and non‐target related mechanisms. The potential implications of such findings with regard to dose selection for biologics in GC are also discussed.

**TABLE 1:** Summary of PKs and E‐R information for biologics tested in GC

Target	Molecule	Approved indication	Dose per non‐GC indication	Dose tested for GC	Patient status in pivotal clinical trial in GC	Difference in exposure for PK in GC vs. other solid tumors	Covariates on PKs	E‐R tested in GC?	E‐R at tested dose	Covariates related to E‐R	Impact on dose	Approved in GC
HER2	Trastuzumab^3^	HER2‐positive breast cancer; HER2‐positive metastatic G/GEJ adenocarcinoma	q.w. Loading: 4 mg/kg Maintenance: 2 mg/kg or q3w Loading: 8 mg/kg maintenance: 6 mg/kg	ToGA study: Loading: 8 mg/kg Maintenance: 6 mg/kg q3w Post market Heloise study: Loading: 8 mg/kg maintenance: 6 mg/kg or Loading: 8 mg/kg maintenance: 10 mg/kg	ToGA (NCT01041404): first line locally advanced, recurrent, or metastatic GC/GEJ adenocarcinoma. HELOISE (NCT01450696): first line human EGFR2‐positive metastatic GC/GEJ adenocarcinoma.	Predicted steady‐state AUC, C_max_, and C_min_ is 30–40% lower than MBC	Body weight; prior gastrectomy; albumin levels; ethnicity; alkaline phosphatase levels	Yes	HER2‐positive GC/GEJ: Patients with PD have lower C_min_,_ss_ compared to patients with SD or those with objective response Overall impact reflected in a small difference in the probability of response (46% ORR in lowest quartile of exposure of 55%–64% in the three highest quartiles of exposure. Transferred to OS)	Risk factors (ECOG PS, history of gastrectomy, Asian ethnicity, number of metastatic sites, and level of HER2 overexpression)	FDA recommended investigation of whether shortened OS within the quartile of patients with lowest C_min_ is due to poor prognosis or low trastuzumab exposure. Post market HELOISE study confirmed maintenance dose increase associated with higher trastuzumab concentrations, no increased efficacy and no new safety signals.	Yes
	Pertuzumab^4^, ^8^	HER2‐positive MBC; neoadjuvant and adjuvant treatment of HER2 positive EBC	q3w Loading: 840 mg maintenance: 420 mg	Loading and maintenance: 840 mg q3w or Loading: 840 mg maintenance: 420 mg q3w	Jacob (NCT01774786): first line (HER2)‐positive metastatic GC/GEJ cancer Joshua (NCT01461057): locally advanced or metastatic HER2‐positive of GC/GEJ adenocarcinoma	Steady‐state C_min_ on day 43 were 37% lower than MBC	Albumin levels; lean body weight	Yes	HER2‐positive GC/GEJ: No differences in OS across cycle 1 pertuzumab C_min_ or cycle 5 pertuzumab C_min_,_ss_ quartiles	Asian ethnicity, number of metastatic sites, and level of HER2 overexpression	Change dose from 840/420 mg in BC to 840 mg q3w in GC	No
	Trastuzumab emtansine^7^, ^9^	HER2‐positive MBC; adjuvant treatment of HER2‐positive EBC	q3w 3.6 mg/kg	2.4 mg/kg q.w.; 3.6 mg/kg q3w	Gatsby (NCT01641939): second‐line HER2‐positive, advanced GC	Observed steady‐state AUC, C_max_, and C_min_ is 20–60% lower than MBC	Body weight; serum HER2 shed ECD concentration; tumor burden	No	HER2‐positive BC: after accounting for baseline risk factors, E‐R for efficacy, demonstrated increases in T‐DM1 exposures are related to better efficacy (OS, PFS, ORR). E‐R for safety identified inverse trend for grade 3 or worse hepatotoxicity. No E‐R relationships identified for thrombocytopenia.^a^	NA	The higher dose: 2.4 mg/kg q.w. was tested in GC	No
	Trastuzumab deruxtecan^5^, ^6^	3 L unresectable or metastatic HER2‐positive breast cancer; 2 L locally advanced or metastatic HER2‐positive GC or GEJ adenocarcinoma	q3w in BC 5.4 mg/kg	1.6 mg/kg; 3.2 mg/kg; 5.4 mg/kg; 6.4 mg/kg; 8.0 mg/kg	DS8201‐A‐J202 (NCT03329690): second‐line+ advanced HER2+ GC/GEJ adenocarcinoma	N/A	Tumor size; country; sex; age; body weight; formulation; albumin levels; total bilirubin; aspartate aminotransferase^a^	No	HER2‐positive BC: significant E‐R relationship for ORR, slight trend of higher probability of PFS with higher C_min_; positive trend for higher AUC with higher risk of any grade ILD; no E‐R for nausea, diarrhea and platelet count decrease.^a^	No covariates were significant for exposure‐ORR relationship	Different dose approved in AGC (6.4 mg/kg q3w) vs. MBC (5.4 mg/kg q3w) patients	Yes
VEGF	Ramucirumab^10^, ^11^	2 L advanced or metastatic GC or GEJ adenocarcinoma; 1 L and 2 L metastatic NSCLC；2 L mCRC； HCC	8 mg/kg q2w; 10 mg/kg q2w; 10 mg/kg q3w	8 mg/kg q2w	REGARD (NCT00917384): second line and third line RAINBOW (NCT01170663): second line advanced GC/ GEJ adenocarcinoma.	No PK difference reported across indications	Body weight; albumin levels	Yes	GC/GEJ patients Higher ramucirumab exposures (i.e., model‐predicted C_min_ values after the first dose) associated with longer median OS, PFS, and greater toxicity	Baseline factors associated with reduced OS and PFS (REGARD): presence of peritoneal metastasis, ECOG PS of 1 or greater, and location of primary tumor (GEJ)	E‐R relationship seen in REGARD and RAINBOW led FDA to request post marketing commitment trial to test the hypothesis that higher doses of ramucirumab could be beneficial with a manageable toxicity profile	Yes
	Bevacizumab^12^, ^13^	mCRC, NSCLC, cervical cancer; glioblastoma, RCC; ovarian cancer; HCC	5 mg/kg q2w; 10 mg/kg q2w; 7.5 mg q3w; 15 mg/kg q3w	7.5 mg/kg q3w	AVAGAST (NCT00548548): first line – histologically confirmed, unresectable, locally advanced or metastatic GC/GEJ adenocarcinoma	CL is ~50% higher in AGC.	Body weight; albumin levels; gastrectomy	No	mCRC patients: survival proportional to the magnitude of exposure^a^	NA	NA	No
PD1	Pembrolizumab^14^	Melanoma; NSCLC; HNSCC; cHL; PMBCL; UC; MSI‐H or dMMR; GC; GEJ; cervical cancer: HCC; MCC; RCC; CRC; Endometrial carcinoma; TMB‐H cancer; cSCC; TNBC	i.v. infusion: 200 mg q3w or 400 mg q6w; 2 mg/kg (up to 200 mg) q3w for pediatrics	200 mg q3w	KN‐061 (NCT02370498): second‐line advanced GC/GEJ cancer KN‐181 (NCT02564263): second line advanced esophageal cancer; KN‐062 (NCT02494583): first‐line with advanced GC/GEJ adenocarcinoma; KN‐059 (NCT02335411): 3L+ advanced GC/GEJ adenocarcinoma	The CL is similar to NSCLC, which was 14.5% lower than melanoma	Sex, eGFR, albumin levels, tumor burden, prior ipilimumab therapy, and ECOG‐PS on CL; sex, albumin levels, and ipilimumab prior therapy status on Vc^a^	No	Melanoma and NSCLC patients: Lack of dose or exposure dependency in pembrolizumab OS between 2 and 10 mg/kg^a^	Catabolic activity as a marker of disease severity is potentially a covariate on efficacy vs. a direct PK‐related impact	No difference in dose	Yes
	Nivolumab^15^	Melanoma, NSCLC, malignant pleural mesothelioma, RCC, cHL, SCCHN, UC, CRC, HCC, esophageal cancer, GC, GEJ cancer, and esophageal adenocarcinoma	240 mg q2w; 360 mg q3w; 480 mg q4w; pediatric: 3 mg/kg q2w	CheckMate649: 360 mg q3w or 240 mg q2w; ATTRACTION‐2: 3 mg/kg q2w	ATTRACTION‐2 (NCT02267343): patients with 2 L+ advance GC CheckMate649 (NCT02872116): first line, non‐HER2 positive GC, regardless of PD‐L1 expression	Baseline CL is 33% higher than NSCLC	Body weight; eGFR, albumin levels; LDH; prior gastrectomy; tumor size; ECOG PS; sex; race; tumor type (GC/GEJC, or other cancer)	No	NSCLC patients: No dose or exposure‐efficacy/safety relationship has been identified across the dose range of 3–10 mg/kg q2w^a^	PD‐L1 expression level did not impact OS	No difference in dose	Yes

Abbreviations: AGC, advanced gastric cancer; AUC, area under the curve; BC, breast cancer; cHL, classical Hodgkin lymphoma; CL, clearance; *C*_max_, maximum concentration; *C*_min_, minimum concentration; CRC, colorectal cancer; cSCC, cutaneous squamous cell carcinoma; EBC, early breast cancer; ECOG PS, Eastern Cooperative Oncology Group performance status; E‐R, exposure‐response; FDA, US Food and Drug Administration; GC, gastric cancer; GEJ, gastroesophageal junction; HCC, hepatocellular carcinoma; HER2, human epidermal growth factor receptor 2; HNSCC, head and neck squamous cell cancer; MBC, metastatic breast cancer; mCRC, metastatic colorectal cancer; MCC, Merkel cell carcinoma; MSI‐H or dMMR, microsatellite instability‐high (MSI‐H) or mismatch repair deficient (dMMR) solid tumors; NA, not available; NSCLC, non‐small cell lung cancer; ORR, overall response rate; OS, overall survival; PD, progression disease; PD1, programmed death‐receptor 1; PD‐L1, programmed death‐ligand 1; PFS, progression free survival; PK, pharmacokinetic; PMBCL, primary mediastinal large B‐cell lymphoma; RCC, renal cell carcinoma; SCCHN, squamous cell carcinoma of the head and neck; SD, stable disease; TMB‐H, tumor mutational burden‐high cancer; TNBC, triple‐negative breast cancer; UC, urothelial carcinoma; Vc, central volume of distribution; VEGF, vascular endothelial growth factor; VEGF‐A, vascular endothelial growth factor A; VEGFR2, vascular endothelial growth factor receptor 2.

^a^ Covariates assessment/E‐R analysis was done in other indications not GC.

## COMPARISON OF THE PKS OF BIOLOGICS IN TREATMENT OF GC WITH OTHER SOLID TUMORS

### Anti‐HER2 antibodies/antibody‐drug conjugates

The frequency of HER‐2 overexpression in gastric and gastroesophageal cancer (GC/GEJC) ranges from 4.4% to 53.4%. To date, four approved anti‐HER2 protein therapeutic agents have been evaluated clinically in GC trials: trastuzumab, pertuzumab, trastuzumab emtansine, and trastuzumab deruxtecan. Both trastuzumab and trastuzumab deruxtecan were approved after demonstrating significant clinical benefit in patients with GC. Trastuzumab and pertuzumab are humanized mAbs targeting the HER2 receptor. Prior published population PK (PopPK) analysis suggested lower trastuzumab PK exposure (30%–40% lower in area under the curve [AUC], maximum concentration, and minimum concentration [C_min_]) in patients with advanced gastric cancer (AGC) compared with patients with metastatic breast cancer (MBC) when receiving the same dosing regimen.^3^ A similar trend was observed for pertuzumab.^4^ Trastuzumab deruxtecan is an ADC that combines a humanized anti‐HER2 antibody backbone with a topoisomerase I inhibitor payload through an enzymatically cleavable peptide‐based linker. Initial PopPK analysis of trastuzumab deruxtecan suggested no statistical significance for tumor type when evaluated as a covariate effect, which included ~ 40 patients treated for GC.^5^ However, the approved doses of trastuzumab deruxtecan for MBC and metastatic GC (MGC) of 5.4 mg and 6.4 mg i.v. q3w, respectively, resulted in similar drug exposure despite differing dose amounts, which indirectly implies different PK characteristics in patients with GC.^6^ Trastuzumab emtansine is another ADC with trastuzumab conjugated to the highly potent cytotoxic agent DM1. PopPK analysis suggested lower trastuzumab emtansine exposure (−18% in mean AUC and −6.1% in mean C_min_) in patients with HER2‐positive AGC compared to patients with HER2‐positive MBC following a 2.4 mg/kg q.w. regimen.^7^

Lower drug exposure for anti‐HER2 antibodies/ADCs in GC compared to breast cancer (BC) may impact patient outcomes and thus hold implications towards dose selection. For example, a positive exposure‐response (E–R) relationship for efficacy was observed in trastuzumab phase III studies in GC, which led to a post‐market requirement (PMR) from the US Food and Drug Administration (FDA) to evaluate a different trastuzumab dosing regimen in GC. Although the PMR study (HELOISE) showed a lack of apparent efficacy benefit with higher trastuzumab dose/exposure, this example presents a cautionary tale which highlights the importance of appropriately interpreting positive E–R relationships that may arise due to confounding of PK and disease status.^2^, ^3^ To compensate for the lower exposures observed in GC as compared with other indications, other molecules have adopted higher doses in GC to ensure adequate exposure. For example, a two‐fold higher dose was selected for the phase III study in AGC to ensure pertuzumab or trastuzumab emtansine exposures in AGC were at least as high as that of MBC in order to maintain efficacious concentrations. Unfortunately, even with a higher dose, clinical read‐outs of both molecules in AGC were negative.^8^, ^9^ For trastuzumab deruxtecan, a higher dose was approved for MGC (6.4 mg) versus MBC (5.4 mg).^6^

### Anti‐VEGF antibodies

Angiogenesis is an important process in the progression and metastasis of solid tumors, including gastric adenocarcinoma. Both ramucirumab and bevacizumab, which target VEGF pathways, were tested in patients with GC. Ramucirumab is a human VEGF receptor 2 antagonist with the first indication approved in GC/GEJC. Using a PopPK method with balanced sample size across four to five cancer types, “cancer indication” was not a significant covariate for ramucirumab PK parameters, indicating the PK characteristics of ramucirumab are largely similar for patients across cancer types.^10^ Interestingly, a positive E‐R relationship for efficacy was observed in the ramucirumab phase III studies in GC, which, like the aforementioned case for trastuzumab, similarly led to a PMR from the FDA to assess if patients with GC could benefit from a higher dose.^11^ Bevacizumab binds to VEGF and prevents the interaction of VEGF to its receptor. It has been approved in several solid cancer types; however, it failed in the phase III AVAGAST trial as a first‐line treatment for GC.^2^ Unlike ramucirumab, bevacizumab showed a significantly lower PK exposure in patients with advanced GC and an approximate 50% increase in CL compared with other solid tumors using post hoc PopPK analysis.^12^ It was reported that bevacizumab showed strong positive correlation between trough concentration and survival in metastatic colorectal cancer.^13^ If the E‐R relationship observed in metastatic colorectal cancer is applicable to GC, it is possible that higher doses of bevacizumab may be needed in GC in order to achieve comparable exposure as other indications, which in turn may lead to improved efficacy. However, given the E‐R relationship of bevacizumab has not been characterized in patients with GC, it remains to be determined if a higher dose of bevacizumab would improve the survival benefit in GC.

### Anti‐PD1 antibodies

The PD‐1/PD‐L1 pathway plays an important role in inhibiting immune responses and promoting self‐tolerance, thus preventing the immune system from attacking tumor cells. Blockage of the interaction of PD‐L1 with the PD‐1 receptor can prevent cancer from evading the immune system. Both pembrolizumab and nivolumab block PD1 and are FDA‐approved for treatment of GC. The time‐dependent PopPK model for pembrolizumab showed comparable parameter estimates between patients with GC and non‐small cell lunc cancer (NSCLC), with a 14.5% increase in CL compared to melanoma.^14^ Similarly, in patients with GC/GEJC, nivolumab exposure was reported to be lower relative to the reference in NSCLC, as a result of ~33% greater baseline nivolumab CL in the GC/GEJC population. Consistent with other tumor types, nivolumab CL in patients with GC/GEJC decreased over time.^15^ Given a flat E‐R relationship for efficacy and safety over a wide range of doses/exposures, the small decrease of exposure in GC (~15%–30%) was not expected to compromise clinical efficacy benefit. Thus, despite lower exposure in GC, the same dose used for other solid tumors is currently applied for GC. Trials with anti‐PD‐L1 agents in advanced GC are ongoing. Limited, preliminary data to date suggests a standard dose of anti‐PD‐L1 agents used in other indications may also be adequate for patients with GC/GEJC, despite lower expected exposure in GC/GEJC.

## POSSIBLE MECHANISMS FOR INCREASED CLEARANCE IN GASTRIC CANCER TARGET‐MEDIATED INCREASED CLEARANCE

Target‐mediated drug disposition (TMDD) is a common elimination pathway for biologics occurs when the drug binds with high affinity to its target, resulting in target‐specific elimination. Because it is a product of receptor binding, TMDD represents a saturable process of the target and is most often observed through non‐linear PK patterns which become more pronounced at low mAb concentrations where total CL is most affected. With increased mAb concentration, other nonspecific CL mechanisms predominate.

Although tumor target level related factors (e.g., tumor size and prior gastrectomy) are often reported as significant covariates in PopPK analysis,^3^, ^5^, ^7^, ^12^, ^15^ the overall contribution of TMDD to the observed trend of lower biologic exposure in GC is considered to be negligible based on several considerations. First, although tumor burden related factors were reported as significant covariates in PopPK analysis, it is unlikely to explain the PK difference across indications as the total tumor burden and target expression in GC are not expected to be consistently higher than other indications. Studies showed that the serum HER2 ECD in metastatic breast cancer (median [SD]: 363 [96] μg/L) was much higher than in GC (median of 10.5 μg/L ranges [4.2–190.2 μg/L]). Serum HER2 levels have a good correlation with tissue HER2 status, which indicates HER2 expression in GC would actually be lower than patients with MBC.^16^ Serum VEGF level was similar between GC and other solid tumors.^17^ Similar baseline tumor size was also observed among different solid tumors in pembrolizumab trials and was indicated as an independent prognostic factor for overall survival.^18^ Moreover, higher CL/lower exposure in GC has been observed across different targets, indicating this phenomenon is not limited to a particular target or pathway. In addition, the drug concentration of the selected dose of these agents usually exceeded the target level such that binding would be saturated to achieve maximum efficacy. This is largely a product of wide safety margins for modern biologic agents that permits dosing to a level at or near the plateau for clinical activity. At such high dose levels, TMDD contributes minimally to total CL, and thus mAbs often exhibit linear PK at clinically approved doses. It is likely that TMDD may only account for a small amount of drug CL and would not influence the overall elimination nor have a clinically meaningful impact on PK. Hence, rather than a marker for increased TMDD, a correlation of baseline tumor burden and biologic PK, may be a general proxy of aggressive/advanced disease and other underlying confounders.

## NON‐TARGET SPECIFIC INCREASED CLEARANCE

### Hyper‐catabolism and cachexia

Cancer cachexia is a multifactorial process, which is characterized by high prevalence of rapid weight loss, hypoalbuminemia, and an acute systemic inflammatory response. Presence of cachexia is generally considered a poor prognostic factor for GC outcome and is specifically associated with decreased survival and worsened quality of life. Incidence and prevalence of cancer‐associated cachexia are heterogeneous across cancer indications and vary depending on tumor type and stage. A report based on two independent studies estimated cachexia prevalence for GC at ~87%, a relatively high value which exceeds estimates across most other common solid tumors.^19^

An acute inflammatory response which may coincide with increases in systemic inflammation, such as IL‐6 and c‐reactive protein (CRP), is thought to trigger the pro‐catabolic state that leads to increased systemic catabolism of endogenous proteins. This also results in clinically observable muscle‐wasting in patients with advanced disease and cachexia. Serum CRP has been reported as abnormally high in ~38% of patients with GC and is considered a potential independent prognostic factor for metastatic GC outcomes.^20^ Moreover, chronic inflammation caused by infection with Helicobacter pylori and autoimmune gastritis is an established primary risk factor of GC development.

Hypoalbuminemia is a prevalent comorbidity in cachexia, and albumin levels have shown to explain PK variability in PopPK analyses of the majority of molecules in GC treatment (Table 1). The PKs of biologic agents and albumin are thought to be surrogates for the overall protein catabolism rates and associated recycling mechanisms of neonatal Fc receptor (FcRN), which is critical for regulating lysosomal degradation of albumin and other IgG‐based antibodies. Strong support for the involvement of cancer cachexia and influence on biologic PKs comes from a recent publication by Castillo et al.^21^ where the elevated catabolic CL of mAbs in cachectic humans was replicated in tumor‐bearing, cachectic mice. In two well‐characterized mouse models of cancer cachexia, the tumor‐bearing, cachectic mice showed significantly higher CL of pembrolizumab compared with tumor‐free mice without cachexia. The authors further demonstrated these increases in catabolic CL within cachectic mice coincides with reduction in Fc gamma receptor and transporter expression, which codes FcRN. Critical to the interpretation of this study, the authors focused on the impact of cachexia on the general catabolic drug CL and avoided potential confounding by target‐mediated CL pathways. This was accomplished by using a humanized anti‐PD‐1 agent pembrolizumab, which interacts with murine FcRn, but does not bind murine PD‐1.

### Gastric protein leakage

Protein‐losing enteropathy (PLE) is a condition in which the loss of proteins through the gastrointestinal (GI) tract exceeds the synthesis of proteins by the body and is often associated with low serum protein. PLE has been observed in more than 60 different conditions, including nearly all GI diseases (e.g., Crohn's disease, celiac, Whipple's, and intestinal infections). In conditions causing inflammation and erosions of the GI tract, such as GC with local GI inflammation caused by infection with helicobacter pylori and autoimmune gastritis, the mucosal permeability increases, leading to excessive leakage of serum proteins into the gut. Additionally, increased lymphatic pressure and lymphatic obstruction, which are commonly associated with gastric malignancy, can also increase leak of lymph into the GI tract and lead to protein loss.^22^ Direct relationships between PLE and mAb PKs have been demonstrated in murine models. A quantitative relationship between the CL of fecal alpha‐1‐antitrypsin (A1AT), a biomarker for PLE and the CL of 8C2, a murine IgG1 mAb was obtained via PopPK modeling, indicate PLE is associated with higher mAb clearance.^23^ Recently, a clinical study reported that the infliximab was detected in the fecal samples in majority of the patients with ulcerative colitis and the fecal concentrations of infliximab was higher in non‐responders than responders, indicate that loss of infliximab through the intestinal mucosa could be a mechanism that influence infliximab exposure.^24^ Levitt et al.^25^ suggested that PLE as one of the mechanisms for hypoalbuminemia with increased albumin GI clearance, which may also be applicable to mAbs. In addition, PLE is also commonly observed as a comorbidity in patients with GC and may be one of the factors that impact mAb PK. The role of PLE in reducing the exposure of biologic agents is likely significant and underappreciated in GC as it may be confounded by other intrinsic and extrinsic factors. Further clinical evaluation is needed to assess the impact of PLE on drug exposure and treatment response in patients with GC.

## CLINICAL PHARMACOLOGY CONSIDERATION AND FUTURE DIRECTIONS

In this review, we show that seven out of the eight presented biologics (e.g., mAbs or ADCs) exhibit faster CL and lower drug exposure in GC compared with other solid tumors. Although there are limitations for this exercise, as the comparisons can be impacted by the sample size, variable analysis methods, and differences arising from cross trial comparisons, this phenomenon appears consistent across different treatments and is likely not target‐dependent.

Possible mechanisms for faster CL of biologics in GC are discussed. The potential contribution of target‐mediated CL to the lower exposures in GC is believed to be limited. We propose two alternative mechanisms that may be responsible for faster CL in GC: hyper‐catabolism of endogenous and exogenous proteins due to cancer cachexia/systemic inflammation observed frequently in GC and/or gastric protein leakage due to local inflammation at the GI tract. It is conceivable that assessment of mechanism‐related clinical biomarkers (e.g., CRP, albumin, and A1AT fecal CL) as covariates of PopPK may shed light on the contribution of the two mechanisms and relative influence on biologic PKs in GC.

**FIGURE 1:**
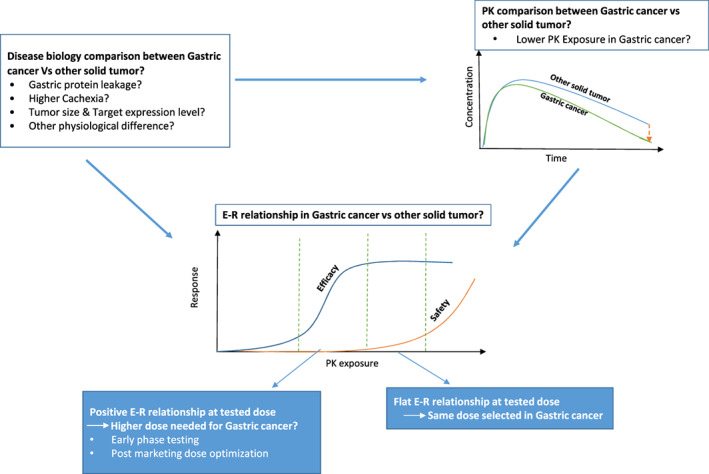
Clinical pharmacology considerations for dose selection in patients with gastric cancer. E‐R, exposure‐response; PK, pharmacokinetic

In light of these observations, it is important to note that not every molecule assessed in this review requires a different dose in GC versus other solid tumors even though lower exposure was observed. In fact, among the eight molecules discussed, only three are administered at a different dose for patients with GC compared with other solid tumors (e.g., trastuzumab deruxtecan, trastuzumab emtansine, and pertuzumab). The potential implication of the apparent lower exposure in GC versus other solid tumors on clinical efficacy and dose selection may vary with the molecule of interest. If an mAb or ADC has a wide therapeutic window with a clinically approved dose at the plateau of dose/exposure‐efficacy response, a small decrease in exposure may not matter in the context of the safety and efficacy of the molecule (Figure 1). Thus, the same dose used in other solid tumors can be applied for GC, as exemplified by pembrolizumab and nivolumab. On the other hand, if the clinically approved dose is located along a steeper portion of the dose/exposure‐efficacy response curve, assessing the benefit and risk profile of a higher dose in AGC may be needed (e.g., trastuzumab deruxtecan; Figure 1). However, for a molecule (e.g., an ADC) with a narrow therapeutic window, increasing dose in AGC may not always be feasible due to potential unacceptable toxicities at the higher dose. In such a case, therapeutic drug monitoring (TDM) and individualized dosing could be considered. If the molecule exhibits large interindividual PK variability and variability observed in response is mainly driven by PK variability, TDM could be an advanced approach to reduce the interindividual PK variability to achieve the target exposure needed for efficacy. Alternatively, if population PK analysis shows that baseline patient covariates (e.g., A1AT, CRP, and albumin) contribute to the large portion of the PK variability, individualized dosing based on these covariates could be considered.

In conclusion, from this review of biologic agents in GC, there is an apparent trend that exposure may be lower in GC as compared with other solid tumors at the same dose. The overall benefit–risk (such as the E‐R) should be considered when selecting the dose to maximize the benefit while minimizing safety risk.

### AUTHOR CONTRIBUTION

All authors contributed to the data collection, hypothesis generation, literature collection, and writing of the manuscript.

### FUNDING INFORMATION

No funding was received for this work.

### CONFLICT OF INTEREST

The authors declared no competing interests for this work.
